# A Retrospective Cohort Study of Neuroendoscopic Surgery versus Traditional Craniotomy on Surgical Success Rate, Postoperative Complications, and Prognosis in Patients with Acute Intracerebral Hemorrhage

**DOI:** 10.1155/2022/2650795

**Published:** 2022-08-05

**Authors:** Yong Li, Senyuan Yang, Xiaobin Zhou, Runlong Lai, Dianhui Tan

**Affiliations:** Department of Neurosurgery, First Affiliated Hospital of Shantou University Medical College, Shantou 515041, China

## Abstract

**Objective:**

A case-control study was adopted to explore the effect of neuroendoscopy compared with traditional craniotomy on the success rate, postoperative complications, and prognosis of patients with intracerebral hemorrhage (ICH).

**Methods:**

The clinical data of 106 patients with ICH treated in our hospital from March 2019 to June 2021 were collected and analyzed retrospectively and divided into two groups according to different treatment methods. The patients who were cured by craniotomy were in the control group (*n* = 53), and those who received neuroendoscopic surgery were in the research group (*n* = 53).The clinical efficacy of patients was compared, and the cognition and daily living ability were evaluated by the Trier cognitive assessment scale, limb motor function score, and activity of daily living scale. The National Institutes of Health Stroke scale (NIHSS) and Glasgow coma scale (GCS) were used to compare the neurological function of the two groups before and after treatment, and the Glasgow outcome scale (GOS) and disability rating scale (DRS) were adopted to evaluate the functional prognosis. The simplified Fugl-Meyer motor function score was adopted to evaluate the patient's limb function, the Montreal cognitive assessment scale was adopted to evaluate the patient's cognitive function, the Barthel index score was adopted to evaluate the daily living ability of patients, and the treatment of patients was recorded.

**Results:**

In comparison with groups, the effective rate of treatment in the research group was higher, and the difference between groups was statistically significant (*P* < 0.05). Regarding the surgical indicators, the hospital stay, intraoperative blood loss, postoperative residual blood flow, and total hospital stay in the research group were remarkably lower, the hematoma clearance rate in the research group was remarkably higher, and the difference between groups was statistically significant(*P* < 0.05). After operation, the KPS scores indicated a gradual upward trend, and those of the research group were higher at 1 month, 2 months, and 3 months after operation. The Barthel index scores were compared. After treatment, the Barthel index scores increased. In comparison with the two groups, the Barthel index scores of the research group were higher at 1 month, 2 months, and 3 months after surgery, and the difference between groups was statistically significant (*P* < 0.05). The NIHSS, GCS, and DRS scores were compared. After treatment, the NIHSS, GCS, and DRS scores were decreased. In comparison with the two groups, the NIHSS, GCS, and DRS scores of the research group were remarkably lower, and the difference between groups was statistically significant (*P* < 0.05). With regard to the cognitive and physical function recovery after treatment, the MoCA score and Fugl-Meyer score of the research group were remarkably higher, and the difference between groups was statistically significant(*P* < 0.05). The quality of life scores was compared. After treatment, the quality of life scores decreased. In comparison with the two groups, the scores of physiological function, psychological function, social function, and healthy self-awareness of the research group were lower, and the difference between groups was statistically significant (*P* < 0.05). The incidence of postoperative complications in the research group was significantly lower than that in the control group, and the difference between groups was statistically significant (*P* < 0.05).

**Conclusion:**

Compared with conventional craniotomy, neuroendoscopic surgery can remarkably reduce the operation time and blood loss, enhance the hematoma clearance rate, and have a better prognosis, which is more conducive to the recovery of postoperative neurological function, life activities, and quality of life of patients.

## 1. Introduction

Intracerebral hemorrhage (ICH) refers to primary or nontraumatic rupture of cerebral blood vessels [1]. It is an acute cerebrovascular disease with acute onset, morbidity, disability, and mortality. It seriously harms people's physical and mental health and brings heavy economic burden to the family and society. There are 1.2–1.8 million new stroke patients in my country every year, with an annual prevalence rate of 2.50/100,000 and a mortality rate of 1.224/100,000, ranking second among all causes of death [2]. About 3/4 of the survivors have varying degrees of severity including incapacity to work, of which more than 40% are severely disabled. The morbidity and mortality of cerebrovascular diseases increased significantly in old age. With the trend of population aging, people over the age of 60 in China will account for more than 10% of the total population [3]. Cerebral hemorrhage directly damages the local brain tissue and destroys the nerve conduction pathway.

Hypertensive ICH accounts for 15% of all strokes, with rapid onset, rapid progression, and high mortality and disability rates [4]. According to the results of epidemiological surveys in my country, the annual incidence of hypertensive ICH is 50–80/10,000 people, and the age is mostly concentrated in 50–70 years old, of which 60–69-year-old patients account for 30.98% of the total, and the percentage of men is more than women [5]. ICH often occurs suddenly during emotional agitation or activity, accompanied by sudden symptoms of focal neurological impairment. After onset, it often reaches its peak within a few minutes to hours, often accompanied by elevated blood pressure, headache, vomiting, meningeal irritation sign, and disturbance of consciousness. According to some data, the mortality rate at 1 month after cerebral hemorrhage is extremely high, about 40%, and only 12%∼39% of survivors do not have disabilities [6]. Another study shows that, in the survivors of cerebral hemorrhage, about 20% of patients have recurrent cerebral hemorrhage. The loss of direct medical expenses in my country caused by cerebral hemorrhage every year is nearly 20 billion, which brings pain to patients, seriously affects their life and work, and increases the economic burden of their families, bringing a heavy burden to the operation of national and social medical funds [7]. The onset of cerebral hemorrhage is sudden, the disease changes rapidly, and the consequences of delayed treatment are serious. Therefore, early diagnosis and treatment have become the focus of the treatment of cerebral hemorrhage. Starting the stroke channel as soon as possible and the cooperation between neurology and surgery can remarkably reduce the mortality and disability rate of patients.

Cerebral edema is the main complication of ICH, and its occurrence and development are the key factors leading to the deterioration of ICH [7, 8]. The main methods for clinical treatment of intracerebral hemorrhage are conservative medical treatment and surgical treatment, and there is no clear reason to prove the absolute advantage of these two methods. However, from the point of view of the treatment effect and treatment process, the use of surgical methods is beneficial to clear the hematoma, enhance the ischemic problem, and reduce the injury of the involved hematoma to the patients, so it is more in line with the development of medicine. Melmed and other industry researchers have summarized clinical experiments and treatment data and found that neuroendoscopy can effectively remove the intracerebral hematoma, reduce the amount of bleeding during treatment, and enhance the treatment effect and prognosis [8]. The American Heart Association guidelines also point out that surgery has a positive effect on removing cerebral hematoma and the optimal treatment time is within 12 hours of onset. At present, the clinical treatment of cerebral hemorrhage mainly includes traditional cranial hematoma evacuation, burr hole hematoma drainage, microsurgical hematoma evacuation, and neuroendoscopic minimally invasive hematoma evacuation. Classical craniotomy with craniotomy for hematoma debridement has a large exposed area of brain tissue, which is prone to cause unnecessary damage, and the operation time is 5 times that of endoscopic surgery. Moreover, the hematoma clearance rate is low, and the patient needs to be injected with urokinase repeatedly to maintain his life after surgery, which easily increases the probability of intracranial infection. Microsurgery also requires stretching of the brain tissue, and excessive stretching increases the likelihood of cerebral ischemia and cerebral edema. In the context of medical development, neuroendoscopic minimally invasive surgery has become the main clinical surgical method for the treatment of cerebral hemorrhage due to its small trauma, less blood loss, and ability of completely removing brain edema. Clinical practice shows that the skin incision using neuroendoscopic surgery is only 3–4 cm and the blood loss is much lower than other surgical methods. This surgical method uses a transparent guide with a diameter of 1 cm to quickly establish a minimally invasive surgical channel, determine the location of the hematoma cavity, and determine the degree of hematoma removal, with good results [9]. In order to verify the therapeutic effect of neuroendoscopic surgery, the author retrospectively analyzed the effect of grouping treatment of hypertensive intracerebral hemorrhage patients who underwent surgery in our hospital.

## 2. Patients and Methods

### 2.1. Normal Information

The clinical data of 106 patients with ICH treated in our hospital from March 2019 to June 2021 were collected and analyzed retrospectively and divided into two groups according to different treatment methods. Patients cured by craniotomy were included in the control group (*n* = 53), and patients who underwent neuroendoscopic surgery were included in the research group (*n* = 53). In the control group, the age ranged from 64 to 85 years old, with an average of 66.12 ± 6.35 years old, including 20 males and 16 females. In the research group, the age ranged from 65 to 84 years, with an average of 67.08 ± 6.79 years, including 19 males and 17 females. The general data of patients were not statistically significant. This study was permitted by the medical ethics committee of our hospital, and all patients noticed informed consent.

Selection criteria: (1) according to the Diagnostic Essentials of all kinds of Major Cerebrovascular Diseases in China 2019 formulated by Neurology Branch of Chinese Medical Association [10], the following accords with the diagnostic points of ICH: (2) acute onset, (3) admission within 48 hours after onset, (4) brain CT or MRI showing blood foci, (5) participating in relevant examination and treatment, and (6) complete clinical data.

Exclusion criteria: (1) complication with abnormal function of important organs, such as heart, liver, kidney, and lung, (2) mental illness, (3) the possibility of death in a short time, (4) patients with excessive hematoma, cerebral hemorrhage in other parts, or brain pain that need bone flap decompression, (5) complication with infectious diseases, and (6) incomplete clinical data.

### 2.2. Treatment Methods

Conventional craniotomy: for patients with supratentorial cerebral hemorrhage, the surgical incision avoids important intracranial vessels and functional areas, makes a horseshoe-shaped incision about 4 cm long, and grinds a bone window with a drill and a milling cutter. The intracerebral hematoma was removed as much as possible under the microscope, and the skull was routinely closed. For patients with preoperative brain herniation or patients with severe brain swelling after removal of the hematoma during surgery, decompressive craniectomy should be given. For patients with bleeding into the ventricle, unilateral or bilateral ventricle drainage should be performed. For patients with infratentorial ICH, the patient's side is prone. According to the location of the hematoma cavity, the posterior median or paramedian surgical incision is performed. After routine craniotomy, corticotomy is adopted to reach the hematoma cavity. The maximum amount of intracerebral hematoma is removed under the microscope. Ventricular drainage should be performed first in patients with hemorrhage or hydrocephalus.

Neuroendoscopic surgery: German Snake brand 0 degrees, 30-degree neuroendoscopy and a corresponding set of TV monitoring systems, good lighting system, and microdevices corresponding to the operation are used. According to the CT level with the largest amount of hematoma, bypass the important arteriovenous vascular area or functional areas such as sensory and motor as the surgical route. After determining the surgical incision, make a straight incision about 3 cm in length, then drill the hole with a drill, and form a 2 cm × 2 cm bone window with a milling cutter. In this study, a 5 mL BD syringe was modified into a rigid transparent mirror sheath, and the sheath core was a catheter of appropriate size.

### 2.3. Observation Indicator

#### 2.3.1. Efficacy Evaluation Criteria

The curative effect was evaluated by the scoring standard of neurological impairment of stroke established by the fourth National Conference on Cerebrovascular Diseases in 1995. The National Institutes of Health Stroke scale [11] (NIHSS) was used to evaluate the degree of neurological impairment in patients with intracerebral hemorrhage. (1) Basic recovery: NIHSS score reduction ≥91%; (2) markedly effective: 46% ≤ IHSS score reduction <90%; (3) effective: 18% ≤ NIHSS score reduction ≤45%; (4) ineffective: NIHSS score reduction <17%. Note: efficacy calculation formula: (NIHSS score before treatment − NIHSS score after treatment)/NIHSS score before treatment × 100%. The total effective rate = (the number of Basic recovery cases+ the number of markedly effective cases+ the number of effective cases)/the total number of cases × 100%.

#### 2.3.2. Collection of Surgical Indicators

The surgical indicators (operation time, intraoperative blood loss, hematoma clearance rate, total hospital stay, and postoperative residual blood volume) were observed. The hematoma volume before and after operation was measured and calculated by 3D-SIicer software, hematoma clearance rate = (preoperative hematoma volume-postoperative hematoma volume)/preoperative hematoma volume × 100%.

#### 2.3.3. Barthel Index

The Barthel index [12] was adopted to evaluate the daily living ability of the patients before and after the intervention, with a total score of 100 points. The higher the score, the stronger the daily living ability.

#### 2.3.4. Assessment of the Degree of Neurological Deficit

Before treatment and after 8 weeks of treatment, the neurological function of the two groups of patients was recorded, and the neurological deficit was scored by the National Institute of Health Stroke scale (NIHSS) [13], which included the level of consciousness, gaze, visual field, upper limb movement, lower limb movement, ataxia, facial paralysis, sensation, language, dysarthria, neglect, and distal motor function. The higher the score, the more serious the neurological damage of the patient.

#### 2.3.5. Coma Level Assessment

Glasgow coma scale (GCS) [14] was used to evaluate the coma degree of patients, including eye opening, language, and exercise, with a full score of 15. The lower the score, the more severe the coma.

#### 2.3.6. Functional Prognostic Assessment

The Glasgow outcome scale (GOS) [15] was adopted to assess the degree of disability of the patients, which was assigned into death (grade I), vegetative state (grade II: long-term coma, open eyes, and periodic eye opening-awake), severe disability (grade III: inability of taking care of themselves, conscious but with severe mental and physical disabilities), moderate disability (grade IV: ability of taking care of themselves in daily life and of engaging in some daily activities in specialized environments), and good recovery (grade V: return to normal life, self-care, but there may be minor neurological or pathological defects).

#### 2.3.7. Cognitive Function Assessment

Cognitive function recovery was assessed using the Montreal Cognitive Assessment (MoCA) [16], with 11 items, each with a score of 0 to 30, with higher scores indicating better cognitive function.

#### 2.3.8. Motor Function Assessment

The recovery of motor function was assessed by the Fugl-Meyer score [17], with a score ranging from 0 to 100. The higher the score, the better the motor function.

#### 2.3.9. Quality of Life Score

Quality of life scale includes four subscales, namely, physical, psychological, social, and health self-perception, a total of 29 items; the scale's Cronbach's alpha coefficient is 0.79–0.91. The scale uses a 1–5 scale; the lower the score, the higher the satisfaction.

### 2.4. Statistical Analysis

Statistical software SPSS22.0 was adopted to process data, count data were presented as *n*, %, and rank sum test was adopted for comparison of prognosis; measurement data was presented as $\bar{x}\pm s$, and *t* test was performed. *P* < 0.05 was considered statistically significant.

## 3. Results

### 3.1. Comparison of Treatment Effects

**Figure 1:**
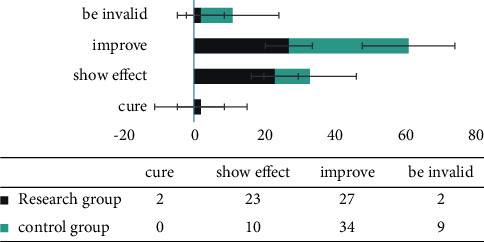
Comparison of the treatment effects of the two groups.

First of all, we compared the therapeutic effects, and the success rate was 100.00%. In the research group, 2 cases were cured, 23 cases were markedly effective, 27 cases were effective, 2 cases were ineffective, and the treatment effective rate was 96.23%. In the control group, 0 cases were cured, 10 cases were markedly effective, 34 cases were effective, 9 cases were ineffective, and the effective rate was 83.02%. In comparison with groups, the effective rate of the research group was higher, and the difference between groups was statistically significant (*P* < 0.05). All results are indicated in Figure 1.

### 3.2. Comparison of Surgery-Related Indicators of Patients

We compared the surgical indicators. The length of hospital stay, intraoperative blood loss, postoperative residual blood flow, and total hospitalization days in the research group were remarkably lower, the hematoma clearance rate in the research group was remarkably higher, and the difference between groups was statistically significant (*P* < 0.05). All results are indicated in Table 1.

### 3.3. Comparison of Postoperative KPS Score

We compared the postoperative KPS score. The research group was followed up to 3 months after operation, and 0 cases were lost. The control group was routinely followed up to 3 months after operation, and 0 cases lost follow-up. There was no significant difference before operation (*P* > 0.05); the KPS score increased gradually after operation, the KPS score at 1 month, 2 months, and 3 months after operation in the research group was higher than that in the control group, and the difference between groups was statistically significant (*P* < 0.05). All the results are indicated in Table 2.

### 3.4. Barthel Index Score Comparison

We compared the Barthel index scores. Before surgery, there was no significant difference (*P* > 0.05); after treatment, the Barthel index scores of patients increased. In comparison with the groups, the Barthel index scores of the research group were higher at 1 month, 2 months, and 3 months after the operation, and the difference between groups was statistically significant (*P* < 0.05). All results are indicated in Table 3.

### 3.5. Comparison of NIHSS, GCS, and DRS Scores

We compared the NIHSS, GCS, and DRS scores. Before treatment, there was no significant difference (*P* > 0.05); after treatment, the NIHSS, GCS, and DRS scores of patients were decreased. In comparison with the two groups, the NIHSS, GCS, and DRS scores of the research group were remarkably lower, and the difference between groups was statistically significant (*P* < 0.05). All results are indicated in Table 4.

### 3.6. Comparison of Cognitive and Physical Function Recovery of Patients after Treatment

We compared the recovery of cognitive and physical function after treatment. In comparison with the two groups, the MoCA score and Fugl-Meyer score of the research group were remarkably higher, and the difference between groups was statistically significant (*P* < 0.05). All results are indicated in Table 5.

### 3.7. Quality of Life Score Comparison

We compared the quality of life scores. Before treatment, there was no significant difference (*P* > 0.05); after treatment, the quality of life scores of patients decreased. In comparison with the two groups, the physical function, psychological function, social function, and healthy self-cognition scores of the research group were lower, and the difference between groups was statistically significant (*P* < 0.05). All results are indicated in Table 6.

### 3.8. Comparison of Postoperative Complications

**Figure 2:**
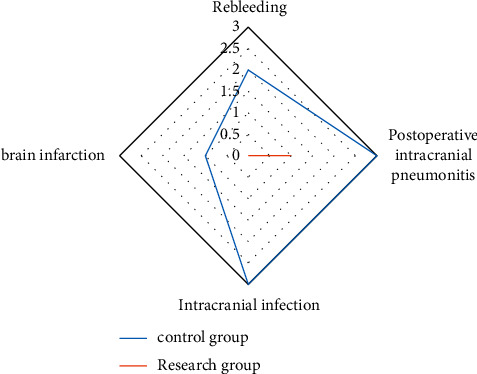
Comparison of postoperative complications between the two groups.

We compared the postoperative complications. In the research group, 1 patient developed intracranial gas after operation, and the total incidence of postoperative complications was 1.89%. In the control group, there were 2 cases of recurrent hemorrhage, 3 cases of intracranial gas accumulation, 3 cases of intracranial infection, and 1 case of cerebral infarction. The total incidence of postoperative complications was 16.98%, the incidence of postoperative complications in the research group was remarkably lower, and the difference between groups was statistically significant (*P* < 0.05). All the results are indicated in Figure 2.

## 4. Discussion

The incidence of ICH has a gradually increasing trend. It poses a great threat to the health of people over 60 years old. ICH can cause acute mass effect, destroy surrounding brain tissue, and often lead to early death of the patient [18]. The space occupying effect of hematoma is an important cause of primary injury. According to epidemiological investigation and analysis, the incidence of cerebral hemorrhage accounts for 10% and 30% of stroke, and primary cerebral hemorrhage with hypertension accounts for 70% of spontaneous cerebral hemorrhage, of which 80% has a higher incidence; it also has high mortality (40% and 60%), high disability rate (50% and 85% survivors), and high recurrence rate [19]. Hypertensive ICH is mainly concentrated in the elderly (over 50 years old), but it shows a younger trend in recent years. The mortality and disability rate of hypertensive ICH are closely related to the location and volume of hemorrhage. With the same volume of hemorrhage, the survival rate of lobar hemorrhage is higher, and the mortality increases linearly with the increase of bleeding volume [20]. In 2000, Montes et al. reported that the 30-day mortality rate of patients with cerebral hemorrhage was about 35%–50%, half of them died within 2 days after onset, only 20% of the patients recovered after 6 months, and a few of them had the ability of living independently [21]. With the aggravation of the aging population and the irregularity of diet and life in our country, the incidence of cerebral hemorrhage is increasing year by year, which is a serious threat to people's health.

Medical treatment is the basic treatment of hypertensive intracerebral hemorrhage, and it is still the first choice for less bleeding, conscious consciousness, and mild neurological dysfunction. Diener et al. believe that the secondary damage after ICH is more serious than the injury caused by hemorrhage itself, so surgical removal of hematoma may be an effective method for the treatment of hypertensive ICH, but there is no statistical significance between STICHI phase response and medical conservative treatment based on the surgical study of ICH. The latest phase II study shows that surgical intervention for superficial hematoma has a better prognosis, indicating that reduction of scratching on brain tissue is effective [22]. It can enhance the effect of operation. Pasi et al. studies have indicated that the time window for stopping bleeding exceeds the previous understanding that bleeding stops within 30 minutes [23]. The hematoma continues to expand due to continuous bleeding and rebleeding, and the mechanism is not clear. Peng et al. believed that the inflammatory cascade reaction led to physiological hemostatic dysfunction, resulting in the destruction of the blood-brain barrier and finally led to the expansion of hematoma [24]. It changed the traditional view that single reactive edema led to the deterioration of early nervous system symptoms and signs and indicated the necessity and importance of early surgical intervention. In addition to the space occupying effect, the toxic effect of hematoma also leads to brain damage. Qureshi and many other animal experiments have confirmed that the coagulation cascade reaction occurs and prothrombin is activated and transformed into thrombin, which leads to brain edema due to neurotoxicity [25]. Yang and Shao believe that the decomposition products of hematoma cause inflammatory reaction around hematoma, destroy the blood-brain barrier, produce inflammatory response, aggravate brain edema, lack local blood and oxygen supply, and induce brain cell apoptosis [26]. The decomposition of hematoma produces free radicals, attacks DNA, and causes oxidative damage to the brain.

At present, there are many schemes for the treatment of hypertensive intracerebral hemorrhage, and surgical treatment of intracerebral hemorrhage has unique advantages: clearing hematoma under direct vision, alleviating space occupying effect, reducing the pathophysiological effect of cytotoxic products after decomposition of blood clots on surrounding tissues, and reducing mortality and disability rate. From the point of view of pathophysiology, surgery may be a better choice for the treatment of cerebral hemorrhage. Early craniotomy for evacuation of hematoma has no obvious clinical significance because of severe trauma and conservative prognosis and internal medicine. With the development of technology, the mode of operation has gradually developed to minimally invasive, the concept of surgery has gradually undergone important changes, and the effect of surgery has gradually been widely recognized in clinical practice.

At present, the choice of surgical indications is different. Yang G thinks that the patients with worsening condition, late cerebral hernia, blood pressure, and respiration need drugs and machine maintenance is not ideal in medical and surgical treatment, so conservative treatment is recommended [26]. Gui et al. think that the amount of bleeding is small, the mind is clear, no matter which kind of treatment is adopted, the outcome is very good, and conservative treatment is recommended [27]. There is a great controversy about the moderate amount of bleeding in 30 ml 60 ml and what kind of treatment. At present, the indications accepted by most doctors are as follows: (1) there is a certain degree of disturbance of consciousness or neurological symptoms, cerebral hernia has not yet formed, or early cerebral hernia should be actively treated; (2) the hemorrhage in the cerebellum is close to the brainstem. Unless the clinical symptoms are mild and the amount of bleeding is small, surgical treatment is the only effective treatment. When cerebellar hemorrhage exceeds 10 ml, there are surgical indications; (3) surgical treatment is not recommended for patients with brainstem hemorrhage, and surgical exploration can be given if vascular malformations and aneurysms are considered; (4) the curative effect of surgery is remarkable in theory, but it cannot be completely confirmed in clinic for the recovery of nerve function. The choice of operation needs to be considered, and whether to operate or not should be combined with the wishes of the family members.

The main purpose of surgery for patients with hypertensive ICH is to stop bleeding, remove hematoma, reduce intracranial pressure, and prevent and reduce a series of secondary pathological changes after hemorrhage. In addition, it can also improve the survival rate and quality of life of patients and promote the prognosis of patients. The disadvantages of craniotomy for evacuation of hematoma are large incision, large trauma, and long operation time, which is not conducive to the recovery of neurological function.

Since Auer first reported the endoscopic treatment of intracranial hemorrhage in 1985, neuroendoscopic technology has made great progress in the past 30 years and has been gradually adopted when treating intracranial tumors and hemorrhage. In this study, the advantages of neuroendoscopy were obtained by comparing the curative effect of neuroendoscopy and routine craniotomy group. In this study, by comparing the efficacy of the neuroendoscopy group and the routine craniotomy group, it was concluded that the neuroendoscopy group had the advantage of less trauma, and the diameter of the trauma caused by it was only 1.5 cm∼2 cm. There were no repeated traction, compression, attraction, and other forms of damage to the important brain tissue around the hematoma, so as to reduce the postoperative reaction and enhance the prognosis of patients. Rennert et al. through the retrospective analysis of 23 cases of endoscopic treatment and 20 cases of microwindow craniotomy when treating cerebral parenchyma hemorrhage found that the prognosis of endoscopy group was remarkably better than craniotomy group [28]. Sun and other prospective studies compared the functional independence assessment (FIM) scale score, Barthel index score, and MP index at 6 months after operation [29]. It is concluded that neuroendoscopic surgery is more beneficial to the recovery of neurological function than craniotomy.

The results indicated that the effective rate of the research group was higher than the control group; the hospitalization time, intraoperative blood loss, postoperative residual blood flow, and total hospitalization days of the research group were remarkably lower than the control group; the hematoma clearance rate of the research group was remarkably higher than the control group, and the KPS scores increased gradually. The KPS score and Barthel index of the research group were higher than the control group at 1 month, 2 months, and 3 months after operation. The scores of NIHSS, GCS, and DRS in the research group were remarkably lower than the control group, while the scores of MoCA and Fugl-Meyer in the research group were remarkably higher than the control group. The scores of physiological function, psychological function, social function, and health self-cognition in the research group were lower than the control group, and there were fewer postoperative complications. The reasons for this can be summarized as follows [29]. (1) Rapid decompression: the process of craniotomy in neuroendoscopic surgery is simplified, so most operations can be completed within 15 minutes for decompression. In completion within 2 h, of course, the proficiency of the operator is also an important factor affecting the operation time. In this study, the operation time of the neuroendoscopy group was remarkably better than the conventional craniotomy group. Some scholars' studies also show that the neuroendoscopy operation is relatively simple, the preparation time is shortened, and the operation time is relatively shortened [30]. (2) Less blood loss during surgery: most operations do not require blood transfusion; (3) there are good deep exposure, wider field of view, and high removal efficiency. The results of this study indicated that the hematoma clearance rate in the neuroendoscopy group (93.5 ± 4.7%) was remarkably better than that in the conventional craniotomy group (90.5 ± 5.2%), similarly to relevant domestic and foreign literature reports. (4) Complete hemostasis: hemostasis of direct vision under the endoscope can reduce the rebleeding rate; (5) the hospitalization cost of patients can also be reduced by reducing complications and hospitalization time.

Neuroendoscopic surgery still has certain limitations in some aspects: (1) the two-dimensional image of the endoscope and the “fish-eye” effect can easily lead to the illusion of the operator, so the operator is required to be more familiar with endoscopy; (2) the “two-dimensional” operation of the thalamus and brainstem is difficult. (3) Neuroendoscopy requires in-depth familiarity and understanding of the local anatomy; (4) internal and (5) special endoscopic surgical instruments are required. (6) The operation requires the cooperation of a professional team; (7) the number of cases is still small, and further research is needed. (8) The operating space and angle of the transparent endoscope sheath are arbitrarily limited. Nishihara et al. invented the transparent endoscopic sheath and achieved a satisfactory hematoma clearance rate [31]. In this study, the self-made transparent endoscope sheath is made from a medical syringe, which can provide a small but large endoscope working channel. A satisfactory hematoma clearance rate was achieved. However, it is found that the relative limitation of surgical space limits the application of other surgical instruments, such as bipolar electrocoagulation. Therefore, the working channel of the endoscope needs to be improved. There are some limitations in this study. First, the sample size of this study is not large and it is a single-center study, so bias is inevitable. In future research, we will carry out multicenter, large-sample prospective studies, or more valuable conclusions can be drawn.

Neuroendoscopy technology is a pair of “wisdom eyes” brought to neurosurgeons by science and technology, and it is a prominent representative of the concept of “minimally invasive neurosurgery,” fast recovery, and low cost. As a new means of diagnosis and treatment, it enhances people's understanding of some diseases and changes the concept of treatment of some diseases. With the continuous improvement of neuroendoscopy technology and the continuous development of surgical instruments, this surgical technique will become more and more mature and will be widely used in clinic.

## Figures and Tables

**Table 1:** Comparison of surgery-related indicators between the two groups [$\bar{x}\pm s$].

Grouping	*N*	Operation time (min)	Intraoperative bleeding volume (ml)	Hematoma clearance rate (%)	Postoperative residual blood volume (ml)	Total hospitalization days (d)
Control group	53	1.24 ± 0.58^a^	326.18 ± 96.83^a^	76.82 ± 7.45^a^	6.35 ± 1.17^a^	16.65 ± 4.53^a^
Research group	53	3.89 ± 1.25^b^	42.24 ± 4.75^b^	92.68 ± 8.87^b^	4.16 ± 0.82^b^	12.43 ± 3.12^b^
*t*/*χ*^2^		14.000	21.322	9.968	11.159	5.585
*P*		＜0.05	＜0.05	＜0.05	＜0.05	＜0.05

Note: the control group before and after treatment, ^a^*P* < 0.05; the research group before and after treatment, ^b^*P* < 0.05.

**Table 2:** Comparison of postoperative KPS scores between the two groups ($\bar{x}\pm s$, points).

Grouping	*N*	Before operation	One month after operation	2 months after operation	3 months after operation
Control group	53	70.31 ± 4.32	75.24 ± 4.76^a^	77.46 ± 4.81^a^	78.12 ± 4.85^a^
Research group	53	71.45 ± 4.16	80.37 ± 5.41^b^	83.48 ± 4.28^b^	85.46 ± 5.96^b^
*t*		1.384	5.183	6.807	6.954
*P*		＞0.05	＜0.05	＜0.05	＜0.05

Note: the control group before and after treatment, ^a^*P* < 0.05; the research group before and after treatment, ^b^*P* < 0.05.

**Table 3:** Comparison of Barthel index scores between the two groups [$\bar{x}\pm s$, points].

Grouping	*N*	Before operation	One month after operation	2 months after operation	3 months after operation
Control group	53	32.54 ± 3.34	47.33 ± 3.43^a^	56.18 ± 5.41^a^	78.31 ± 4.58^a^
Research group	53	33.18 ± 3.75	59.45 ± 4.28^b^	67.63 ± 3.95^b^	86.43 ± 5.75^b^
*t*		0.928	16.087	12.444	8.220
*P*		＞0.05	＜0.05	＜0.05	＜0.05

Note: the control group before and after treatment, ^a^*P* < 0.05; the research group before and after treatment, ^b^*P* < 0.05.

**Table 4:** Comparison of NIHSS, GCS, and DRS scores between two groups [$\bar{x}\pm s$, points].

Grouping	*N*	NIHSS scoring		GCS scoring		DRS scoring	
		Before treatment	After treatment	Before treatment	After treatment	Before treatment	After treatment
Control group	53	30.33 ± 3.16	22.33 ± 3.12^a^	4.36 ± 1.23	8.44 ± 2.48^a^	22.77 ± 3.83	16.18 ± 2.53^a^
Research group	53	30.28 ± 3.53	14.36 ± 2.17^b^	4.74 ± 1.46	13.38 ± 3.41^b^	21.56 ± 3.43	12.45 ± 2.16^b^
*t*		0.077	15.267	1.449	8.529	1.713	8.163
*P*		＞0.05	＜0.05	＞0.05	＜0.05	＞0.05	＜0.05

Note: comparison of control group before and after treatment, ^a^*P* < 0.05; comparison of research group before and after treatment, ^b^*P* < 0.05.

**Table 5:** Comparison of cognitive and physical function recovery between the two groups [$\bar{x}\pm s$, points].

Grouping	*N*	MoCA scoring	Fugl-Meyer scoring
Control group	53	20.17 ± 3.36^a^	58.43 ± 8.77^a^
Research group	53	25.28 ± 3.95^b^	74.33 ± 10.26^b^
*t*		7.174	8.576
*P*		＜0.05	＜0.05

Note: comparison of control group before and after treatment, ^a^*P* < 0.05; comparison of research group before and after treatment, ^b^*P* < 0.05.

**Table 6:** Comparison of quality of life scores between the two groups [$\bar{x}\pm s$, points].

Grouping	*N*	Physiological function		Psychological function		Social function		Healthy self-cognition	
		Before treatment	After treatment	Before treatment	After treatment	Before treatment	After treatment	Before treatment	After treatment
Control group	53	15.36 ± 4.18	13.98 ± 2.37^a^	17.34 ± 3.57	15.13 ± 4.37^a^	18.74 ± 3.05	16.82 ± 2.71^a^	15.63 ± 3.01	13.63 ± 1.56^a^
Research group	53	15.83 ± 4.29	10.42 ± 2.81^b^	16.93 ± 3.49	10.84 ± 1.29^b^	18.56 ± 3.49	12.47 ± 3.89^b^	15.45 ± 3.13	10.32 ± 2.71^b^
*t*		0.571	7.050	0.598	6.854	0.283	6.680	0.302	7.706
*P*		＞0.05	＜0.05	＞0.05	＜0.05	＞0.05	＜0.05	＞0.05	＜0.05

Note: comparison before and after nursing in the control group, ^a^*P* < 0.05; comparison before and after nursing in the research group, ^b^*P* < 0.05.

## Data Availability

The datasets used and analyzed during the current study are available from the corresponding author upon reasonable request.
